# Etymologia: *Borrelia miyamotoi*

**DOI:** 10.3201/eid2008.ET2008

**Published:** 2014-08

**Keywords:** etymologia, Borrelia miyamotoi, bacteria, anaerobic spirochete, ixodid ticks

## *Borrelia miyamotoi *[bə-relʹe-ə miʺa-mo-toʹe]

A genus of gram-negative, anaerobic spirochete bacteria, *Borrelia* was named after French biologist Amédée Borrel. In 1995, Masahito Fukunaga et al. isolated a novel *Borrelia* species and named it *Borrelia miyamotoi*, in honor of Kenji Miyamoto, who first isolated spirochetes from ixodid ticks in Hokkaido, Japan. Human cases of *B. miyamotoi* infection were subsequently found in Russia in 2011 and North America in 2013.
